# Immersive wearable virtual reality for autism: a systematic review of current evidence

**DOI:** 10.3389/fpsyt.2026.1771573

**Published:** 2026-07-27

**Authors:** Chiara Failla, Roberta Minutoli, Paola Chilà, Germana Doria, Ileana Scarcella, Chiara Marraffa, Flavio Corpina, Gaia Roccaforte, Noemi Crifò, Anna Meduri, Giovanni Pioggia, Flavia Marino

**Affiliations:** 1 Institute for Biomedical Research and Innovation (IRIB), National Research Council of Italy (CNR), Messina, Italy; 2 Faculty of Psychology, International Telematic University Uninettuno, Roma, Italy; 3 Department of Cognitive, Psychological Science and Cultural Studies, University of Messina, Messina, Italy; 4 Multi-Specialist Clinical Institute for Orthopaedic Trauma Center (COT), Messina, Italy; 5 Department of Biomedical, Dental and Morphological and Functional Imaging Sciences, University of Messina, Messina, Italy

**Keywords:** autism spectrum disorder, head-mounted displays, immersive technology, virtual reality, wearable virtual reality

## Abstract

**Introduction:**

Immersive and wearable virtual reality (VR) is an emerging technology with growing potential to support assessment and intervention ifor autistic people. The methodological heterogeneity of existing studies limits the interpretation and generalization of current evidence.

**Methods:**

A systematic review with a narrative synthesis was conducted in accordance with the PRISMA guidelines. Electronic searches were performed in PubMed, Scopus, IEEE Xplore, Web of Science, and Google Scholar, identifying studies published between 2015 and August 2025. Twenty-two studies investigating wearable and immersive VR interventions in children and adults with ASD met the eligibility criteria.

**Results:**

The included studies demonstrated that wearable VR interventions may improve social communication, joint attention, emotional regulation, daily living skills, executive functioning, and user engagement. Innovative technologies, including eye-tracking and artificial intelligence-based systems, also enabled objective assessment of gaze behaviour, social interaction, and physiological responses. Nevertheless, the evidence was characterized by considerable methodological heterogeneity, predominantly small sample sizes, limited use of randomized controlled designs, and scarce long-term follow-up, reducing the generalizability of the findings.

**Discussion:**

Wearable VR represents a promising tool for personalized assessment and intervention in ASD. Based on the current evidence, we propose a structured pre-intervention assessment integrating sensory, cognitive, emotional, and VR tolerance profiles to support individualized intervention planning. Future research should prioritize standardized outcome measures, rigorous study designs, and longitudinal investigations to strengthen the clinical translation of VR-based interventions in autism.

## 1 Introduction

Autism Spectrum Disorder (ASD) is a neurodevelopmental disorder characterized by limitations in social communication and interaction, repetitive behaviors, and restricted interests (1). The prevalence of ASD, which typically emerges in the first years of life, has increased significantly over the past two decades. Data from the Autism and Developmental Disabilities Monitoring (ADDM) Network, funded by the U.S. Centers for Disease Control and Prevention (CDC), revealed that the estimated prevalence increased from 1 in 150 children in 2000 to 1 in 44 in 2018 (2), with the most recent CDC report indicating that 1 in 36 children is affected by ASD (3). This upward trend is attributed to multiple factors, including the broadening of diagnostic criteria, heightened public awareness, and more frequent diagnoses among women and adults (4) highlighting ASD as a growing public health concern that requires effective intervention strategies. A core feature of ASD is persistent deficits in social communication and interaction across multiple contexts (1). Children with ASD often experience difficulties initiating and maintaining conversations, sustaining joint attention, and responding appropriately to social cues, which limits their ability to form meaningful relationships (5). Social skills, defined as both verbal and nonverbal behaviors that facilitate mutually beneficial interactions or as abilities that enable independent functioning and enhance quality of life (6–8), are thus critical targets in interventions for this population. Historically, interventions for social skills have included methods such as video modeling, behavioral skills training, social stories, peer- and sibling-mediated interventions, antecedent interventions, manualized curricula, pivotal response training, milieu teaching, naturalistic interventions, reinforcement strategies, script fading, discrete trial training, and self-management (9). However, these approaches face challenges, including the time-intensive creation of diverse instructional scenarios and the difficulty in replicating certain naturalistic situations (10). Research has highlighted that individuals with ASD often demonstrate a strong preference for structured, visually supported content, along with strengths in visual memory and detail-oriented processing (11–16). This receptiveness to digital media has prompted the development of technology-assisted instruction and intervention (TAII), which encompasses the use of common technologies such as computers and mobile devices, as well as advanced tools including virtual reality (VR), augmented reality (AR), mixed reality (MR), and robots (17, 18). VR enables the creation of immersive, interactive, and repeatable environments where distractions can be minimized and scenarios can be carefully controlled. Compared with traditional interventions, VR allows not only the safe simulation of real-life contexts but also the collection of rich behavioral and physiological data—such as gaze patterns, emotion recognition, and affective responses—that are difficult to capture in naturalistic settings (19). VR systems used in autism research range from desktop-based applications and Cave Automatic Virtual Environments (CAVE) to wearable immersive VR devices such as head-mounted displays (HMDs) and VR glasses (20). Wearable devices are particularly promising because they provide high levels of immersion and ecological validity, while remaining relatively portable and accessible. Advances in display technologies have made these devices increasingly affordable and adaptable, allowing for individualized interventions across developmental needs (21, 22). Despite the growing interest in wearable VR interventions for ASD, the evidence base remains fragmented. Existing studies vary widely in their targeted skills, methodological protocols, and device characteristics, making it difficult to establish standardized guidelines or identify the most effective approaches. Therefore, this systematic narrative review aims to map and synthesize the literature on wearable immersive VR devices (including HMDs and VR glasses) used with individuals with ASD. In this review wearable immersive virtual reality was operationally defined as any head-worn technology system that provides users with an immersive or semi-immersive digital experience through a device physically worn on the head, including head-mounted displays (HMDs), smart glasses, and mixed-reality headsets. These systems may deliver fully virtual environments or digitally overlaid content within the real world (augmented/mixed reality), provided that the primary interaction occurs through an embodied, head-mounted interface. Eligible systems were required to enable real-time interaction within the environment using sensory modalities such as head tracking, gaze, hand controllers, or integrated eye-tracking, thereby allowing user engagement with dynamic stimuli in controlled or semi-controlled environments. Non-wearable systems (e.g., desktop-based VR, screen-based simulations, or projection-based environments) were excluded. This definition emphasizes the hardware configuration (head-worn systems) and the level of embodied immersion rather than the specific technological label (VR, AR, or MR), ensuring conceptual consistency across heterogeneous devices used in ASD interventions. Specifically, it seeks to: (1) identify the targeted skill areas, including social communication, daily living skills, cognitive functions, attention, and sensory engagement and (2) examine the intervention protocols applied. By consolidating current evidence, this review provides a clear overview of VR applications in ASD, highlights knowledge gaps, and informs the development of evidence-based, standardized VR interventions tailored to the unique needs of this population.

## 2 Methods

**Figure 1:**
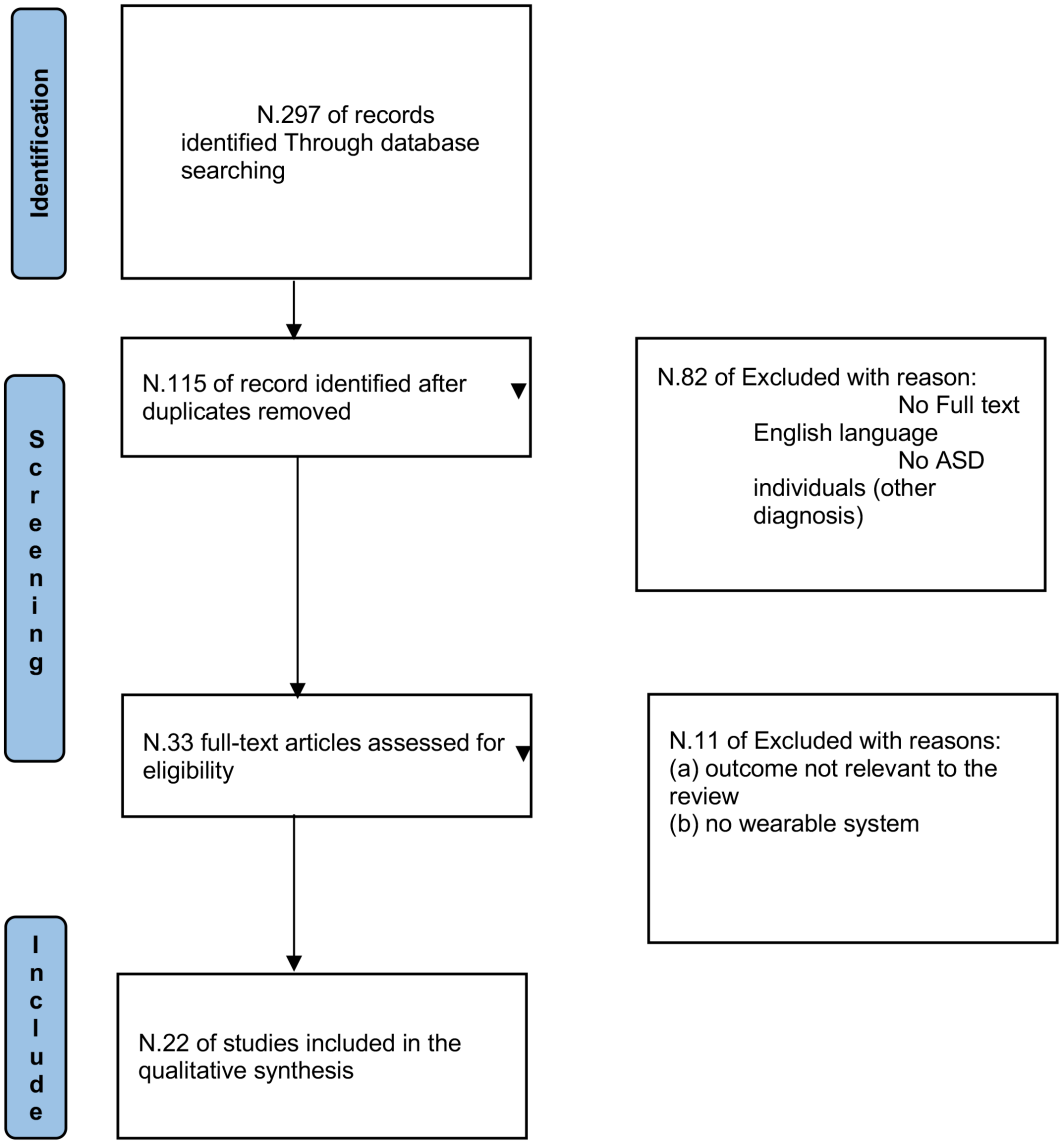
The PRISMA analysis.

This review was planned and conducted as a systematic review in accordance with the PRISMA (Preferred Reporting Items for Systematic Reviews and Meta-Analyses) guidelines (**Figure 1**) (23). Due to the substantial methodological and clinical heterogeneity among the included studies, findings were synthesized narratively. The search was limited to studies published from 2015 to ensure methodological and technological homogeneity. This decision was based on the substantial shift in virtual reality technologies occurring during this period, marked by the emergence and widespread adoption of modern consumer-grade head-mounted displays (e.g., Oculus Rift, HTC Vive, Microsoft HoloLens). Prior to 2015, most VR applications in autism research relied on non-wearable or non-immersive systems (e.g., desktop-based VR or Cave Automatic Virtual Environments), which differ significantly in terms of immersion, interaction modality, and ecological validity. For these reasons, articles published between 2015 and August 2025 were retrieved from electronic bibliographic databases, including PubMed (, Scopus, IEEE Xplore; WoS and Google Scholar. The search strategy combined Medical Subject Headings (MeSH) and free-text terms related to autism spectrum disorder, immersive technologies, and wearable virtual reality systems. The following search string was adapted according to the requirements of each database: (“Autism Spectrum Disorder” OR ASD OR autism) AND (“Virtual Reality” OR VR OR “Immersive Virtual Reality” OR “Head-Mounted Display” OR HMD OR “Wearable System” OR “Wearable Technology” OR “Augmented Reality” OR AR) AND (training OR intervention OR rehabilitation OR assessment OR therapy OR education OR social skills OR daily living skills). Additional manual searches were conducted by screening the reference lists of all eligible articles to identify potentially relevant studies not captured by the database search. Two reviewers (C.F. and A.M.), after removing duplicate records, independently screened titles and abstracts for eligibility. Full-text articles meeting the inclusion criteria were subsequently assessed independently by both reviewers. Any disagreements regarding study eligibility were resolved through discussion and consensus. Inclusion and exclusion criteria were defined *a priori* (1) Inclusion criteria (a) written in English, full-text, peer-reviewed; (b) involving participants with ASD; (c) using head-worn wearable virtual reality systems (2) Exclusion criteria (a) studies using other immersive technologies not involving wearable systems. The eligibility criteria were defined according to the PICOS framework:

- Population (P): Individuals with a diagnosis of ASD, regardless of age, functional level, or setting.
- Intervention (I): Head-worn wearable technologies providing immersive or semi-immersive virtual, augmented, or mixed reality experiences (e.g., head-mounted displays, smart glasses).
- Comparison (C): Studies with or without a comparison/control group were included, as a comparison condition was not required for eligibility.
- Outcomes (O): Studies reporting outcomes related to social functioning, attentional or perceptual processes, emotion or behavior regulation, daily living skills, usability, acceptability, feasibility, tolerability, safety, engagement, or user experience.
- Study Design (S): Experimental studies (RCTs, quasi-experimental), pilot and feasibility studies, observational studies, case series, and case reports were included. Review articles, theoretical works, and protocols were excluded.

No minimum intervention duration was established as an eligibility criterion because the objective of the review was to provide a comprehensive overview of wearable VR applications in ASD, including feasibility, usability, assessment, and intervention studies.

Data were independently extracted by two reviewers using a structured extraction form, including: study characteristics, participant demographics, intervention type, outcomes measured, VR system characteristics, and key findings. Given the diversity of the included studies (n = 22) in terms of design, outcomes, and VR systems a narrative synthesis was employed. Rather than applying *a priori* thematic domains, we used an inductive thematic analysis: after extracting data, we reviewed all studies and derived five thematic categories based on recurring patterns in the functional and social domains targeted by the interventions. These emergent categories are: (1) Social Skills & Emotional Regulation (social communication, joint attention, empathy, affective expression, emotional regulation), (2) Daily Living & Functional Skills (autonomy, practical everyday abilities), (3) Cognitive, Executive, and Learning Skills (planning, working memory, inhibition, higher-order cognitive functions, (4) Sensory Processing & VR Engagement (visual/auditory attention, sensory desensitization, immersion, usability), (5) Assessment & Innovative Technologies (performance monitoring, eye−tracking, AR integration via wearable VR). Within each category, findings were synthesized by comparing the characteristics of the wearable system, the VR tasks, outcome measures, and evidence of feasibility, acceptability, or clinical impact. This inductive, domain-driven synthesis allowed us to map meaningful conceptual clusters in the literature and to highlight methodological gaps and future research directions.

## 3 Results

### 3.1 Study selection

**Figure 1** reported the four phases—identification, screening, eligibility, and inclusion— of the process for the selection of the studies in this qualitative review. During the identification phase, a total of 297 records were retrieved from the electronic databases, including PubMed (n = 39), Scopus (n = 15), IEEE Xplore (n = 47), Web of Science (n = 24), and Google Scholar (n = 172). Then duplicates were removed, resulting in a total of 115 unique records. The screening phase then began, during which 82 articles were excluded because they were either not written in English or did not involve individuals with ASD. This left 33 articles for further assessment. Of these, 11 were subsequently excluded because the virtual systems used were not wearable or the measurements reported were not relevant to the objectives of this review. At the end, 22 studies were included in the systematic narrative review. Data extracted from each article included first author and year of publication, sample size, experimental procedures, target population, study design, outcome measures, and main findings. The complete data extraction table is provided in the **Supplementary Materials**. A condensed summary of the included studies is presented in **Table 1**, which reports the following key domains: study, condition/functional level of participants, VR system/game used, primary motor/social/daily skills targeted, and key outcomes.

**Table 1:** Summary of the included studies.

Study	Condition/functional level of participants	VR system/game used	Primary motor/social/daily skill improved	Key outcome
El-Gohary et al., 2022 (30)	11 children/adolescents with ASD (7–18 y)	Oculus Rift + Leap Motion VR serious game (shopping simulation)	Daily living skills (shopping, money use, social interaction)	Improved task performance and evidence of transfer to real cafeteria setting
Junaidi et al., 2020 (37)	7 adolescents with ASD (13–18 y)	Unity-based HMD VR educational system	Learning skills, instructional comprehension	Feasible and usable; moderate effectiveness but ergonomic and comprehension limits
Samantaray et al., 2024	5 children with ASD + 10 TD	Eye-gaze AR/VR joint attention system	Joint attention, social communication	Feasibility demonstrated; gaze and physiological responses successfully captured
Jyoti et al., 2019 (38)	20 ASD + 20 TD participants	VR joint attention training platform	Joint attention, social cue processing	ASD group showed reduced gaze cue-following vs TD; system detected JA deficits
Alimanova et al., 2022	Children with ASD (4–9 y)	Oculus Quest 2 “Virtual Farm” VR	Social communication, emotion recognition	High engagement; adaptive VR responses increased interaction quality
Herrero & Lorenzo, 2020 (36)	8–15 y children (EG vs CG ASD)	Immersive VR classroom & social scenarios	Social-emotional skills	Significant improvements in social communication, empathy, and emotional regulation
Voss et al., 2019 (40)	40 ASD children (EG) + 31 controls	Superpower Glass wearable + app	Social engagement, emotion recognition	Significant improvements in socialization and facial engagement
Shahab et al., 2021 (34)	5 children with high-functioning ASD	HTC Vive VR music training system	Cognitive, imitation, musical skills	Improvement in musical performance and imitation; feasibility confirmed
Johnston et al., 2020 (35)	6 adolescents with ASD	Oculus Rift auditory VR exposure (SoundFields)	Sensory processing (auditory desensitization)	Reduced anxiety and improved tolerance to auditory stimuli
Dixon et al., 2020 (20)	3 children with ASD	Oculus Rift VR street-crossing training	Safety/daily living skills	Mastery of street-crossing skills with generalization to real-world
Rosenfield et al., 2019 (39)	2 children with ASD + 1 ADHD	Oculus Rift VR shop environment	Social communication	High engagement and feasibility; exploratory evidence only
Adjorlu et al., 2018 (41)	5 young adults with ASD (18–22 y)	HTC Vive VR money management system	Money management skills	Partial improvement in financial task performance
Newbutt et al., 2016 (42)	29 children with ASD	Oculus Rift immersive VR environments	Engagement, spatial presence	High acceptance and engagement with HMD VR
Beach & Wendt, 2016 (43)	2 adolescents with ASD	Immersive VR + real-world training (Second Life)	Social interaction skills	Improved social interaction with transfer to real-world settings
Cheng et al., 2015 (44)	3 children with ASD (10–13 y)	HMD immersive VR social scenarios	Social understanding	Improvements in social behaviors maintained post-intervention

### 3.2 Study and sample characteristics

**Figure 2:**
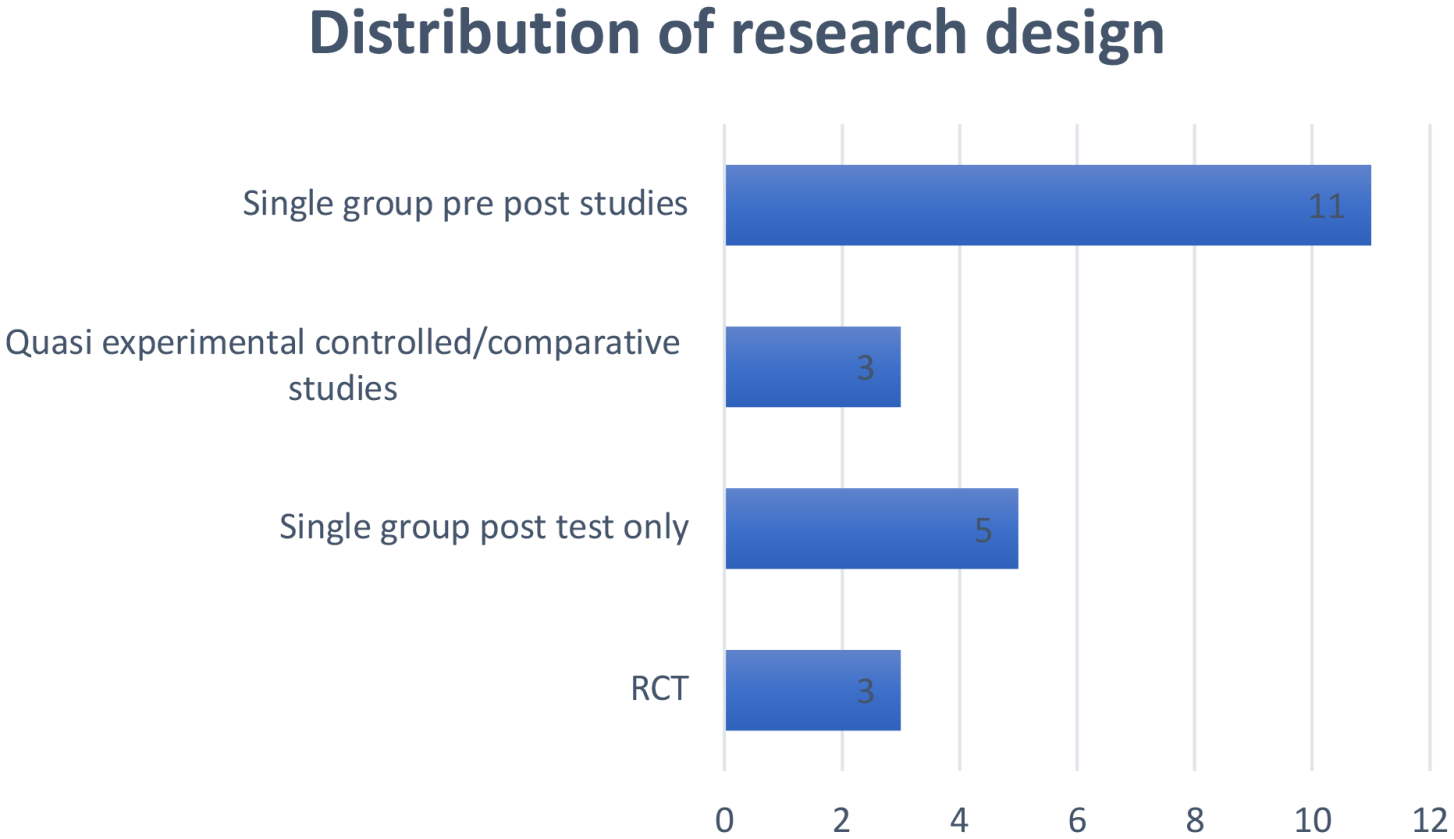
Distribution and type of research design of the studies included in the review.

Study sizes ranged from 2 to 190 participants, with ages of recruited participants spanning from 4 to 53 years. A total of 493 participants were enrolled across the seventeen studies (436 men and 56 women). The predominance of study designs is characterized by single-group, pre–post designs and their variants in fact they represent the 50% of the studies (n=11) included in this review (see **Figure 1**) Specifically, studies by Li Li et al. (2025) (24), Kourtesis et al. (2023) (27), Soltiyeva et al. (2023) (28), Meng & Yeh (2022) (33), Adjorlu et al. (2018) (41), and Johnston et al. (2020) (35), employed standard single-group pre–post designs, while variations including follow-up, maintenance, transfer, or generalization were used in studies by El-Gohary et al. (2022) (30), Mojtaba Shahab et al. (2021) (34), Dixon et al. (2020) (20), Beach & Wendt (2016) (43), and Yufang Cheng et al. (2015) (44). This pattern suggests that researchers in this field frequently rely on within-subject designs to assess intervention effects over time. Randomized controlled trials (RCTs) were less common, only 13% with only three studies—Ip et al. (2022) (32), Catalin Voss et al. (2019) (40), and Herrero & Lorenzo (2020) (36)—using this rigorous experimental approach, indicating a relative scarcity of studies with high internal validity. Only three study, conducted by Jeppesen et al, (2025) (25) Samantaray et al. (2024) (26), and Jyoti at al. (2019) (38), used a quasi experimental controlled/comparative studies and five studies, conducted by Alimanova et al. (2022) (29), Elkin et al. (2022) (31), Junaidi et al. (2020) (37), Rosenfield et al. (2019) (39) Newbutt et al. (2016) (42) are categorized in to the last type of design denominated single group post test only. **Figure 2** shows the global distribution of the research design.

### 3.3 Methodological quality and risk of bias assessment

Methodological quality and risk of bias were assessed using the evaluative framework proposed by Reichow et al. (2011) (45), which was specifically developed to evaluate the methodological rigor of intervention studies in individuals with autism spectrum disorder. Two reviewers (C.F. and A.M.) independently assessed the methodological quality of each included study. Any discrepancies between reviewers were resolved through discussion until consensus was reached. Each study was evaluated across four methodological domains: internal validity risk, measurement bias risk, intervention fidelity risk, and generalizability risk. For each domain, a rating of low, moderate, or high risk of bias was assigned according to predefined criteria adapted from the Reichow framework. The complete risk-of-bias assessment for each individual study is reported in the **Supplementary Material**, where all domain-specific judgments are presented together with the corresponding justifications explaining the rationale for assigning each risk category.

**Figure 3:**
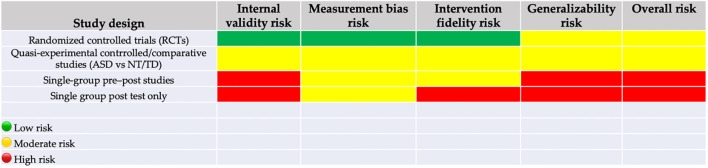
Summary of risk of bias across study designs based on the Reichow et al., 2011 framework.

An overall risk-of-bias judgment was subsequently derived for each study by considering the pattern of ratings across the four methodological domains, reflecting the cumulative methodological limitations and strengths of each investigation. The results of the risk-of-bias assessment are presented at two complementary levels: a detailed study-level evaluation including domain-specific ratings and justifications (**Supplementary Material**), and a summarized overview according to study design category (**Figure 3**), illustrating the distribution of methodological risk across the different types of research designs included in the review.

### 3.4 Target domains of VR interventions in ASD

**Figure 4:**
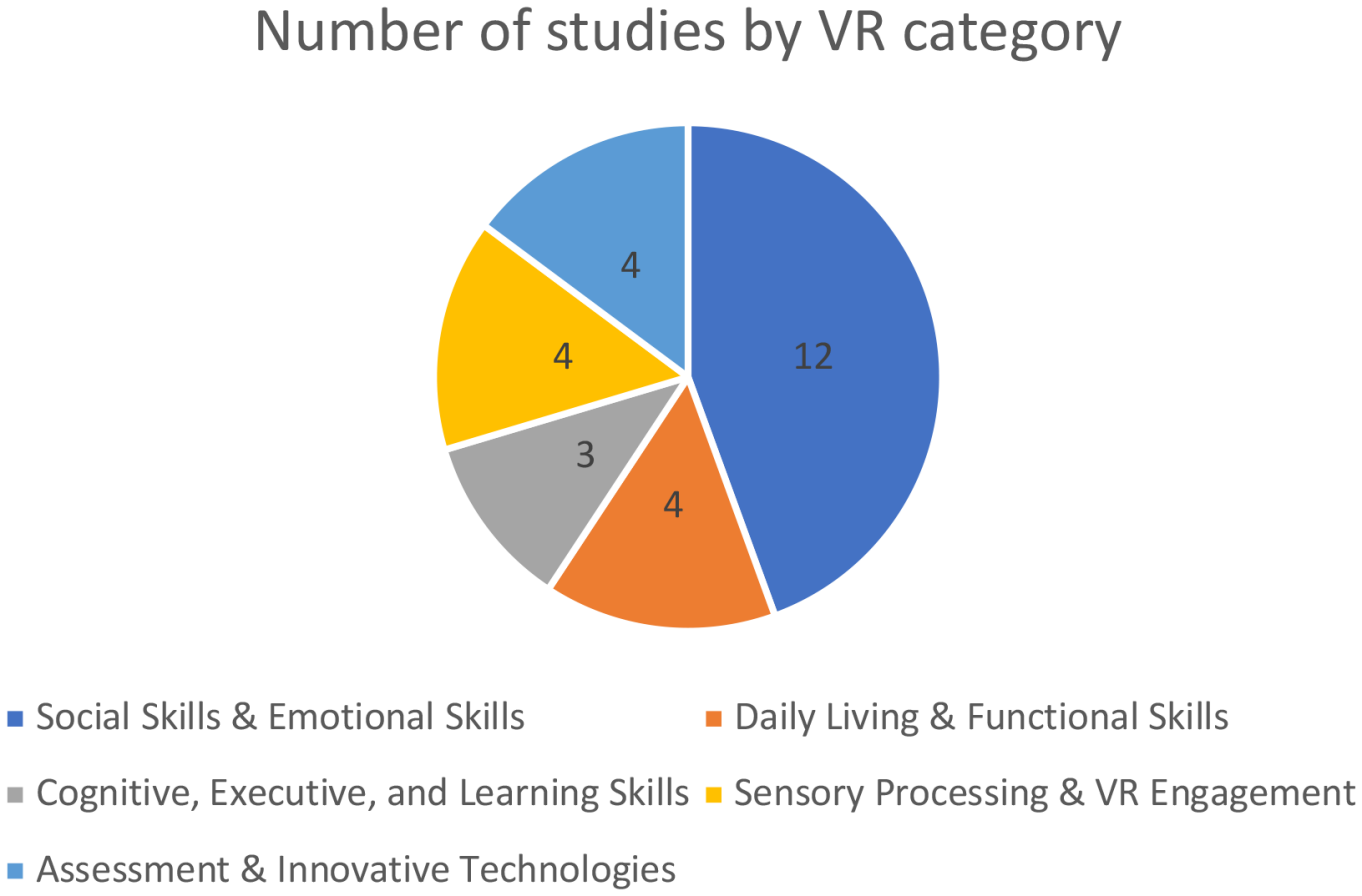
Number of included studies, classified according to the thematic domains identified in the review. Note: studies may be represented in more than one category; therefore, totals across domains are not mutually exclusive.

The studies included in this systematic review examine a wide range of social skills. Identifying which social skills are most frequently addressed is crucial to understanding current trends and gaps in the field. Based on the compiled table, several target domains of intervention using VR for autistic individuals can be identified (see **Figure 4**). These include: Social Skills & Emotional Regulation, focusing on social communication, joint attention, empathy, affective expression, and emotional regulation; Daily Living & Functional Skills, aimed at enhancing autonomy and practical abilities in everyday life; Cognitive, Executive, and Learning Skills, supporting planning, working memory, inhibition, and higher-order cognitive functions; Sensory Processing & VR Engagement, addressing visual and auditory attention, sensory desensitization, immersion, and usability and Assessment & Innovative Technologies, involving the use of VR and wearable devices for performance monitoring, eye-tracking, and AR integration. These categories highlight the diverse potential of VR as a flexible and tailored tool for autism interventions, with each domain targeting specific skill sets and functional outcomes. **Figure 4** illustrates the distribution of the studies across the different domains. Since several VR-based interventions target multiple functional domains simultaneously, some studies are included in more than one category; therefore, domain classification should be considered overlapping rather than mutually exclusive. Specifically, the study by Samantaray et al. (26) contributed to both the Daily Living & Functional Skills and Assessment & Innovative Technologies domains; the studies by Jeppesen et al. (25) and Johnston et al. (35) each contributed to both the Sensory Processing & VR Engagement and Assessment & Innovative Technologies domains, with Johnston et al. (35) additionally contributing to the Social Skills & Emotional Regulation domain; finally, the study by Soltiyeva et al. (28) contributed to three domains (Social Skills & Emotional Regulation, Sensory Processing & VR Engagement, and Assessment & Innovative Technologies). Consequently, twelve studies (26–28, 31, 32, 35, 36, 38–40, 43, 44) focused on social communication, joint attention, empathy, emotional regulation, affective expression, and conflict resolution, forming the Social Skills & Emotional Regulation category. Four studies (20, 24, 30, 41) concentrated on developing practical abilities and autonomy in daily life, including self-care, cooking, money management, street safety, and task execution, forming the Daily Living & Functional Skills domain. Three studies (33, 34, 37) combine cognitive and executive functions (planning, working memory, attention) with educational and experiential learning forming Cognitive, Executive, and Learning Skills. Four studies (25, 28, 35, 42) focused on the management of sensory perception, attention, tolerance to stimuli, and engagement in VR, forming the Sensory Processing & VR Engagement domain. Four studies (25, 26, 28, 35) focused on the use of VR and wearable technologies to measure, monitor, and integrate new assessment tools, forming the Assessment & Innovative Technologies category.

### 3.5 Virtual reality and socio-emotional skills

The first category identified for socio-emotional skills consists of nine studies involving a total of approximately 327 participants, aged between 4 and 52. The interventions focus on the acquisition of socio-communicative skills, emotional reciprocity, and affective regulation, using virtual environments that mimic everyday situations such as school, work, or informal social interactions. Analysis of these studies has highlighted increased engagement, verbal communication, and social tolerance after exposure to VR (26, 28, 36, 43), while others have documented significant improvements in social reciprocity, and affective expression in structured school settings (29, 32). In recent years, the use of other technological tools has also emerged, characterized by platforms ranging from complex systems with integrated eye-tracking and interactive avatars (28) to more adaptive solutions sensitive to the user’s emotions (28) or wearable devices equipped with artificial intelligence, such as Superpower Glass, capable of providing real-time feedback during everyday interactions (40). Studies aimed at adults (27) have demonstrated high usability and acceptability, suggesting that virtual reality can be an effective tool even beyond developmental age (38). A methodologically heterogeneous profile emerges, ranging from randomized controlled clinical trials (27) to pilot and single-case studies (43, 44), up to exploratory qualitative studies (37).

### 3.6 VR and daily living skills

The second domain identified for this review consists of studies that have focused on the use of VR for learning daily living skills in children and adolescents with autism. A literature review from the last ten years reveals three studies involving participants aged 4 to 22, with varying levels of functioning. Li et al. (2025) (24) developed four three-dimensional virtual environments—a cafeteria, a library, a school entrance, and a simulated aquarium—in which children aged 4 to 16 practiced daily activities guided by visual and auditory stimuli, showing significant improvements in practical skills and social communication. El-Gohary et al. (2022) (30), in a single-group pre–post study, reported that a VR-based environment enhanced engagement and supported the acquisition of daily living skills, with evidence of skill transfer to real-world contexts. Adjorlu et al. (2018) (41) used a virtual reality program to train money management skills, involving five adolescents in a three-tiered, progressively more complex program, which led to increased performance and independence during virtual shopping tasks. Dixon et al. (2020) (20) demonstrated the effectiveness of a virtual reality program aimed at road safety, teaching children aged 4 to 10 years how to assess when to cross the road. Participants achieved mastery criteria both in virtual scenarios and in transferring to real-world situations, supporting the generalization of the acquired skills.

### 3.7 Virtual reality and cognitive-learning skills

The third skill domain identified encompasses the macro area of ​​cognitive-learning skills, encompassed by two scientific studies that used immersive technologies to promote situational learning and the acquisition of specific skills. Shahab et al. (2021) (34) implemented a virtual reality music education program for five autistic children, using virtual robots and musical instruments in structured weekly sessions. Results showed improvements in musical performance during the sessions, with a slight decline at follow-up. The integration of an automatic assessment system allowed for scalable and objective measurement of progress. Similarly, Meng Yeh et al., 2022 (33) proposed a virtual reality course titled “An Art Tour with Students” for ten students with high-functioning learning disabilities, aimed at improving social interaction, environmental adaptation, and communication skills. Through four simulated activities in real-world scenarios—from a classroom to a visit to an exhibition, a restaurant, and a park—students participated in eight virtual reality sessions spread over three and a half months. The results showed significant improvements in inappropriate social behavior and adaptation to the environment, confirmed by teacher and parent assessments. The study also identified some operational issues, such as difficulties using controllers and managing the virtual space, which could impact the effectiveness and safety of learning. Junaidi et al. (2020) (37), using a single-group posttest-only design, evaluated the feasibility and usability of a Unity-based VR educational application for autistic adolescents. The intervention showed promising usability and content validity according to teacher evaluations, although participants experienced initial anxiety, fatigue, and ergonomic discomfort.

### 3.8 VR and sensory engagement

The fourth clustering area concerns the use of VR for sensory aspects in the autistic world. We found studies examining how virtual environments can promote improved sensory regulation, social attention, and tolerance to external stimuli. The analyzed studies included approximately 187 participants aged 4 to 52, covering a broad spectrum of cognitive functions. The interventions differed in their use of VR as a tool for desensitization, sensory exploration, or neurobehavioral assessment. Johnston et al. (2020) (35) demonstrated that an interactive VR sound environment (“SoundFields”), based on gradual exposure techniques to anxiety-provoking acoustic stimuli, can significantly reduce auditory anxiety in autistic adolescents by improving sound tolerance and active participation. Newbutt et al. (2016) (42) evaluated the usability and safety of HMDs in a group of adults with varying cognitive levels, highlighting high acceptability and the absence of significant side effects. Participants reported a positive immersive experience and a heightened sense of presence, regardless of their level of intellectual functioning. On the more experimental front, Jeppesen et al. (2025) (25) introduced an innovative approach based on immersive eye-tracking in VR, which allows for the objective measurement of eye fixation patterns during simulated social interactions. The study involved a large sample of adults with ASD (n = 140) and neurotypical controls (n = 50), showing systematic differences in fixation times on areas of interest such as the eyes and mouth, supporting the validity of virtual reality as an ecologically reliable neuropsychological assessment tool. The study by Soltiyeva et al. (2023) (28), while also applicable to social skills, significantly integrates the sensory domain thanks to an adaptive system capable of detecting and responding to the user’s emotions through visual and audio feedback, promoting multisensory and personalized learning.

### 3.9 VR and assessment and technological innovation

The macro-area dedicated to the use of virtual reality and wearable devices as assessment tools and technological innovation includes studies that aim to overcome the limitations of traditional assessment methodologies by introducing objective, automated, and highly ecological approaches to observing social behavior. Overall, the studies analyzed in this area involved 211 participants, covering a wide range of ages and levels of functioning, and proposed the use of advanced technologies such as immersive eye-tracking and AI-based wearable devices. The study by Jeppesen et al. (2025) (25) represents a step toward the objective quantification of social attention through the integration of eye-tracking in immersive VR environments. Involving 140 adults with ASD and 50 neurotypical controls, the researchers analyzed visual behavior during six virtual social scenarios of varying complexity, using a Varjo Aero headset equipped with a high-frequency eye-tracker (200 Hz). The results showed distinctive fixation patterns in participants with ASD, with significant differences in fixation times and attention to socially relevant areas such as the eyes and mouth. This evidence suggests that virtual reality, combined with biometric metrics, can provide precise and replicable behavioral indicators, improving the sensitivity and objectivity of traditional clinical assessments. In parallel, Voss et al. (2019) (40) explored a complementary approach through the use of AI-based wearable technologies, developing the Superpower Glass system as a home-based therapeutic support for children with ASD. The device, based on Google Glass, integrates a mobile application that recognizes the interlocutor’s facial expressions in real time, providing visual and verbal feedback to the child during everyday interactions. In a randomized clinical trial with 71 participants, results showed significant improvement in social skills, particularly in the Vineland Adaptive Behavior Scale socialization scale, compared to the control group receiving ABA therapy alone. The studies included in this section demonstrate how the combination of immersive VR, eye-tracking, and artificial intelligence is redefining the role of digital technologies in autism assessment and intervention. These emerging approaches allow for the collection of objective behavioral data, real-time monitoring of therapeutic progress, and personalized interventions based on each individual’s cognitive and sensory profile.

## 4 Summary of evidence

The 22 studies included in this review highlight how immersive, wearable virtual reality (VR) may represent a promising tool for supporting various developmental dimensions in individuals with autism. Accumulating evidence shows consistent improvements in social and emotional skills, with increased verbal and nonverbal communication, empathy, emotional regulation, and understanding of social norms (27, 28, 32, 36). Interventions targeting daily living skills have demonstrated effectiveness in learning practical skills such as money management, road safety, and domestic independence (20, 24, 41). Studies focusing on sensory engagement have confirmed VR’s ability to reduce hypersensitivity to auditory stimuli and promote attention and familiarity with new environments (32, 37), while the integration of innovative technologies such as eye-tracking and wearable artificial intelligence devices allows for more objective and personalized assessments of social and cognitive performance (25, 40). The positive results obtained in these studies across various intervention areas, however, still present some critical issues: the sample size is typically small, the protocols and results obtained are often heterogeneous, the duration of the interventions is often very limited, and, above all, there is no follow-up to confirm the robustness of the results. Many studies do not assess the generalizability of the skills acquired to real-world contexts or only partially integrate cognitive, social, and sensory aspects. These observations highlight the need to develop standardized and broader protocols capable of comprehensively evaluating the potential of virtual reality as a therapeutic and educational tool for individuals with ASD.

## 5 Limitation

Despite the findings highlighted in the reviewed studies, several methodological and conceptual limitations must be acknowledged. First, most of the included studies were based on small and heterogeneous samples, often composed of individuals with high-functioning ASD or within narrow age ranges, thus limiting the generalizability of the findings to the broader autism population. Only three of the 22 studies included a larger sample size (25, 32, 40) and only three other studies (32, 36, 40) included a control group alongside the experimental group. Therefore, the lack of randomized controlled trials and the predominance of pilot and feasibility studies also reduce the strength of the available evidence and make it difficult to establish causal relationships between virtual reality-based interventions and observed behavioral or cognitive improvements. Few studies included long-term follow-up assessments to assess the maintenance and generalization of skills acquired in real-world settings. Measures of sensory engagement, emotional regulation, and daily functioning were often self-reported or based on qualitative observations, with limited use of standardized and objective outcome measures such as physiological or neurocognitive indicators. Technological and ethical challenges—such as cybersickness, sensory overload, and accessibility for individuals with limited cognitive or motor abilities—remain underexplored and should be systematically addressed in future research. The heterogeneity of outcome measures represents another important limitation when interpreting findings across functional domains. Although several studies reported improvements following VR-based interventions, direct comparison between studies remains challenging because outcomes were assessed using different standardized instruments, researcher-developed tasks, observational coding schemes, and technology-derived metrics. For example, within the social skills domain, some studies evaluated broader constructs such as social communication or adaptive behavior through validated questionnaires, whereas others focused on specific behavioral indicators such as gaze fixation or joint attention response latency. Therefore, conclusions regarding VR effectiveness should be interpreted as domain-specific rather than representing a uniform treatment effect. A major limitation of current VR-based interventions is the limited assessment of real-world generalizability. Although immersive environments allow controlled and standardized evaluation of specific skills, improvements observed within virtual scenarios do not necessarily translate into equivalent changes in naturalistic settings. Only a subset of studies included real-world transfer assessments or follow-up evaluations (20, 30, 41, 43), limiting the ability to determine whether acquired skills are maintained and effectively applied in everyday contexts. These limitations underscore the need for more rigorous, large-scale, and longitudinal investigations that integrate multimodal assessment tools and ecologically valid virtual reality paradigms to better define the efficacy, usability, and long-term impact of immersive technologies in autism intervention.

## 6 Discussion

The studies reviewed in this systematic narrative review demonstrate that VR can promote significant improvements across a wide range of developmental domains, from social-emotional communication and daily living skills to cognitive functioning and sensory regulation. Evidence suggests that wearable VR has the potential to bridge the gap between structured therapeutic environments and real-world experiences by offering immersive, controllable, and ecologically valid contexts for learning and interaction. Consistent with previous reviews of non-wearable VR applications (11, 46, 47), this review confirms that immersive environments can facilitate social engagement and emotional understanding in individuals with ASD. A critical issue emerging from the review is the high level of methodological heterogeneity across studies; this variability compromises comparability between research and complicates the identification of truly effective VR components for autistic populations. Intervention protocols differ substantially in terms of duration, number of sessions, and training context, contributing to high variability in treatment types even within the same intervention domains. Another important consideration concerns the sensory profiles of individuals with ASD. Some individuals may benefit from the structured and visually salient nature of VR, while others may experience discomfort due to brightness, motion, or auditory amplification. Future research should therefore integrate standardized sensory assessments to determine which subgroups of autistic individuals are most likely to benefit from immersive VR, thus allowing for the creation of increasingly individualized protocols. A major challenge identified in studies concerns the generalization of skills acquired through VR to real-life contexts. Although several interventions have reported improvements within VR environments, few have assessed whether these skills transferred to naturalistic settings or persisted over time. This limitation echoes previous concerns that controlled VR scenarios, while ecologically richer than traditional computer-based tasks, may still lack the unpredictability of everyday social interactions (15). Another gap in the literature concerns the integration of VR interventions with healthcare providers, teachers, and clinicians. Most studies have implemented VR in clinical or research settings with minimal involvement of naturalistic stakeholders. This is problematic, given that healthcare provider-mediated interventions are known to improve learning consolidation and generalization in autism spectrum disorders (48). Future interventions should aim to integrate healthcare provider coaching or hybrid models combining VR and naturalistic approaches. Ethical and safety considerations also deserve attention. Although cybersickness has rarely been reported as a major issue, the integration of eye-tracking, behavioral metrics, and physiological sensors in wearable VR raises growing data protection concerns, particularly in pediatric populations. Furthermore, informed consent, distress management, and prevention of over-reliance on virtual environments remain important topics that future research and regulatory guidelines should address (49, 50). Despite the technological capabilities of current head-worn devices, objective measures such as eye-tracking indices, physiological correlates (e.g., heart rate variability, skin conductance), or detailed behavioral analyses are still not widely used in current clinical practice. These modalities offer unique insights into attentional patterns, emotional regulation, and underlying mechanisms of change, and their inclusion could strengthen both theoretical and clinical conclusions (51, 52). Many studies involved fewer than 15 participants and employed pilot or quasi-experimental designs. Robust randomized controlled trials with adequate statistical power, active comparison groups, and longitudinal follow-ups are needed to validate claims of efficacy and inform evidence-based clinical recommendations. Overall, the present review highlights both the potential and the current limitations of wearable immersive VR technologies for autism spectrum disorder. While preliminary findings support feasibility and acceptability and, in some cases, demonstrate significant improvements in targeted skills, the field remains in an early developmental phase. Greater methodological rigor, increased personalization, systematic integration of sensory profiles, enhanced ecological validity, and broader adoption of multimodal outcome assessments will be crucial steps toward establishing VR as a credible and effective tool for neurodevelopmental intervention.

### 6.1 Future directions

Future research in this area should primarily focus on longitudinal studies with follow-ups extending from 6 to 24 months to determine the long-term stability of VR-related gains (53). Furthermore, standardizing intervention protocols and outcome measures is crucial, as there is significant heterogeneity in these future VR interventions (54). Another important aspect concerns the progress in wearable sensors, psychophysiological monitoring, and artificial intelligence, which can support the development of adaptive and personalized VR systems, capable of adapting content based on attentional, behavioral, or physiological responses (55). Another relevant direction concerns broadening the focus of VR interventions beyond social skills, exploring areas such as emotional regulation, executive functions, coping strategies, and independence in daily activities, which remain underrepresented despite strong theoretical justification. Similarly, hybrid intervention models combining VR practice with real-world activities, mediated by educators, parents, or therapists, are proposed to improve skill generalization and real-life applicability (56). Additional concerns include safety, tolerability, and sensory comfort, as some studies report cybersickness, sensory overload, or fatigue, particularly in highly immersive environments (57). It is therefore essential to establish standardized guidelines for monitoring and mitigating adverse effects. Finally, expanding research across different age groups and functional profiles, including very young children, adults, individuals with intellectual disabilities, or with increased sensory sensitivity, remains a crucial step to assess scalability and inclusivity (58). Current findings confirm the potential of VR as an innovative and impactful tool to support social, cognitive, emotional, and adaptive development in autistic individuals.

### 6.2 Proposed pre-intervention assessment battery for VR-based interventions in ASD

Given the promising results reported with wearable and immersive virtual reality interventions for individuals with autism, an important future direction involves the development of a standardized pre-intervention assessment battery designed to assess individual sensory, cognitive, and sensorimotor profiles. Such an assessment would support the identification of predictors of adaptability to virtual reality and optimize the personalization of intervention protocols. Recent studies have demonstrated that sensory and behavioral profiling can identify meaningful subgroups within the autism spectrum, each characterized by distinct adaptive functioning and a different likelihood of benefiting from specific interventions (59). Furthermore, early studies using immersive virtual reality for objective sensory assessment (e.g., visual and tactile modulation tasks) suggest that virtual reality can sensitively capture perceptual responses that are not always evident through questionnaires alone (60). Given evidence that cognitive flexibility is frequently impaired in ASD (61), and that virtual-reality based interventions may improve executive and attentional functions in this population (62), future research should empirically test whether baseline cognitive profiles (e.g., flexibility, attentional control) predict responsiveness and generalization following VR-based social and adaptive training. Combining cognitive and sensory information within a unified predictive framework may therefore enhance VR tailoring and improve both feasibility and long-term outcomes. For these reasons, we propose a four-component pre-intervention assessment model, integrating (see **Table 2**):

**Table 2:** Domains and measures for VR adaptation assessment.

Domain	Standardized assessment tools	Indicators	VR adaptation decision
Sensory processing	Sensory Profile 2; Short Sensory Profile; Adolescent/Adult Sensory Profile	Hyper-reactivity, sensory seeking, auditory/visual sensitivity	Reduce visual complexity, modify sound intensity, introduce gradual exposure, select less immersive scenarios
Sensorimotor & perceptual functioning	Beery-Buktenica VMI; Developmental Test of Visual-Motor Integration; Movement Assessment Battery for Children-2 (MABC-2)	Visuo-motor integration, coordination, balance	Adjust interaction modality (controller vs gaze-based interaction), increase environmental support
Cognitive & executive functioning	NEPSY-II Attention and Executive Functions; Behavior Rating Inventory of Executive Function (BRIEF-2); Trail Making Test; Stroop tasks	Attention control, flexibility, inhibition, working memory	Modify task complexity, pacing, cueing level, cognitive load
Emotional/social functioning	Emotion Regulation Checklist; Emotion Regulation Questionnaire; Reading the Mind in the Eyes Test; Social Responsiveness Scale-2 (SRS-2)	Social motivation, emotion recognition, regulation capacity	Select social scenarios, avatar complexity, feedback strategies
VR tolerance/cybersickness	Virtual Reality Sickness Questionnaire (VRSQ); Simulator Sickness Questionnaire (SSQ); VR exposure trial	Nausea, discomfort, physiological stress	Adjust exposure duration, locomotion, field of view, breaks

1. Sensory Profile Assessment
  Standardized instruments (e.g., Sensory Profile 2) and physiological or behavioral markers to evaluate hypo-/hyper-reactivity, tolerance to visual/auditory stimulation, and regulation across sensory modalities (63).
2. Sensorimotor and Perceptual Processing Evaluation
  Tasks assessing visuo-motor coordination, perceptual thresholds, postural stability, and sensory integration capacities, which have been shown to differ systematically across ASD subgroups and to predict tolerance to immersive stimulation.
3. Cognitive and Emotional Functioning Assessment
  Measures of attention, executive functions, emotion regulation, and social motivation, which influence learning readiness and the capacity to benefit from VR-supported adaptive and social skill training (64).
4. VR Tolerance and Cybersickness Risk Screening
  Pre-exposure questionnaires, brief VR acclimatization tasks, and physiological stress responses to identify early signs of discomfort, sensory overload, or susceptibility to cybersickness in pediatric neurodivergent populations (65).

Implementing such an assessment model would make it possible to predict individual VR suitability, tailor immersive environments to specific sensory–cognitive profiles, reduce adverse experiences, and substantially increase the ecological value of VR as both an assessment and intervention tool. Moreover, integrating pre-intervention profiling into future research protocols will support the identification of moderators of treatment response, enabling the development of more robust, evidence-based clinical guidelines for wearable VR in ASD.

In clinical and educational settings, the proposed assessment battery could be implemented as a stepped decision-making process. Before VR intervention, individuals would undergo a brief multidisciplinary evaluation including sensory, cognitive, emotional, and VR tolerance domains. Results would not be used as exclusion criteria but rather to personalize intervention parameters. For example, individuals showing sensory hypersensitivity could receive shorter exposures, reduced environmental complexity, or gradual habituation protocols, whereas individuals with preserved sensory tolerance but executive difficulties may benefit from increased scaffolding, simplified task sequences, and adaptive feedback.

### 6.3 Safety, ethical, and data privacy considerations in VR-based interventions for ASD

Despite the growing interest in immersive virtual reality and wearable technologies as innovative tools for autism intervention, safety, ethical, and data privacy considerations represent critical aspects for their successful clinical translation. Autistic person, particularly younger children and those with greater cognitive, communicative, or sensory impairments, may present increased vulnerability to adverse responses associated with immersive environments, including cybersickness, sensory overload, anxiety, discomfort, and difficulties in adapting to virtual stimuli. These factors may influence not only the acceptability of VR-based interventions but also the validity of performance outcomes, as reduced engagement or distress may interfere with task completion and learning processes. The implementation of VR interventions should be preceded by a structured assessment of individual characteristics, including sensory reactivity, cognitive readiness, communication abilities, emotional regulation, and tolerance to immersive stimulation. As discussed in the proposed pre-intervention assessment framework, these evaluations should not be considered exclusion criteria but rather tools to support personalization and optimize intervention delivery. Information obtained from baseline profiling could guide the adaptation of relevant VR parameters, including exposure duration, environmental complexity, visual and auditory intensity, interaction modality (e.g., controller-based, gesture-based, or gaze-based interaction), and the level of therapist or caregiver support required during sessions. Observable signs such as avoidance behaviors, withdrawal, agitation, reduced engagement, or increased emotional arousal should be considered relevant indicators for modifying or interrupting VR exposure. Similarly, physiological measures, when available, may provide additional objective information regarding stress responses and adaptation to immersive environments. Beyond immediate safety considerations, the increasing integration of eye-tracking systems, physiological sensors, and artificial intelligence (AI)-based wearable technologies introduces additional ethical challenges related to the collection, storage, and interpretation of sensitive personal data. Eye movements, gaze patterns, physiological responses, and behavioral signatures represent highly informative datasets that may reveal individual characteristics beyond the specific objectives of the intervention. Future VR-based ASD research and clinical applications should incorporate privacy-by-design principles, including secure data storage, anonymization procedures, restricted access to collected information, and transparent communication with families regarding what data are collected, how they are processed, and for what purposes they are used. Integrating pre-intervention profiling, continuous monitoring, and robust data governance strategies may enhance the ecological validity, acceptability, and long-term sustainability of VR applications across clinical and educational settings.

## 7 Conclusion

This systematic narrative review highlights the transformative potential of immersive, wearable virtual reality in interventions with children with ASD. The results show that VR can support multiple developmental areas, from social communication to sensory integration to daily living skills, offering safe, controllable, and motivating contexts that traditional methods often struggle to replicate. Despite these promising results, the field is still in its infancy: many studies have small sample sizes, heterogeneous protocols, and limited generalizability to real-world settings. To address these challenges, future research should not only consolidate standardized protocols and clear outcome metrics, but also adopt more personalized approaches that take into account participants’ individual sensory profiles. Specifically, the proposal of a pre-intervention assessment based on sensory, cognitive, and emotional profiles could guide the personalization of VR programs, improving tolerance, engagement, and the effectiveness of interventions. The integration of biometric, eye-tracking, and artificial intelligence technologies already enables the collection of behavioral and physiological data in real time, offering unique opportunities to tailor virtual reality scenarios to the specific needs of each participant. Looking ahead, the combination of standardized methodologies, detailed sensory assessments, and personalized immersive interventions could pave the way for more effective, environmentally sustainable, and truly person-centered treatments. Wearable virtual reality represents not only an innovative technology but also a powerful tool for redefining clinical and educational practice in autism spectrum disorders, provided that future research continues to integrate objective data, individual profiles, and real-world scenarios to ensure safe, sustainable, and truly transformative interventions.

## Data Availability

The original contributions presented in the study are included in the article/**Supplementary Material**. Further inquiries can be directed to the corresponding author.
