# Supplementary material for: Immersive wearable virtual reality for autism: a systematic review of current evidence
Source: Front Psychiatry. 2026 Jul 27;17:1771573. doi: 10.3389/fpsyt.2026.1771573 (PMC13454810; doi:10.3389/fpsyt.2026.1771573)
Supplement: Supplementary file 1 [file Table1.docx]

| **Study** | **Design** | **Internal validity risk** | **Measurement bias risk** | **Intervention fidelity risk** | **Generalizability risk** | **Overall risk** |
| --- | --- | --- | --- | --- | --- | --- |
| **Li Li et al.,**  **(2025) [24]** | Single Group pre-post test | High risk  **Justification:** single group | Low risk  **Justification:**  Outcomes assessed using validated standardized instruments and/or objective technological measures. | Low risk  **Justification:**  Intervention protocol clearly described | High risk  **Justification:**  Small sample size | High risk |
| **Alberte Cathrine Ehrhardt Jeppesen et al.,**  **(2025) [25]** | Quasi Experimental | Moderate risk  **Justification:**  no randomization or experimental manipulation | Low risk  **Justification:**  Outcomes assessed using validated standardized instruments and/or objective technological measures | Low risk  **Justification:**  Intervention protocol clearly described | Moderate risk  **Justification:**  large sample size reduces detection bias | Moderate |
| **Samantaray et al., 2024** | Quasi experimental | Moderate risk  **Justification:**  absence of randomization and potential confounding between ASD and NT groups limits causal inference despite experimental task structure. | Low risk  **Justification:**  Outcomes assessed using validated standardized instruments and/or objective technological measures | Moderate risk  **Justification:**  standardized task delivery across conditions, but limited reporting of procedural fidelity and system calibration details | High risk  **Justification:**  Small sample size | Moderate high risk |
| **Kourtesis et al., (2023) [26]** | Single Group pre-post test | High risk  **Justification:**  single group | Low risk  **Justification:**  Outcomes assessed using validated standardized instruments and/or objective technological measures. | Low risk  **Justification:** | Moderate risk  **Justification:** | Moderate risk |
| **Soltiyeva et al., (2023) [27]** | Single Group pre-post test | High risk  **Justification:**  single group | High risk  **Justification:**  Outcomes primarily based on qualitative observations, non-validated measures | Low risk  **Justification:**  Intervention protocol clearly described | High risk  **Justification:**  Small sample size | High risk |
| **Alimanova et al., 2022** | Single Group post test only | High risk  **Justification:**  single group | High risk  **Justification:**  Outcomes primarily based on qualitative observations, non-validated measures | High risk  **Justification:**  Limited description of intervention procedures | High risk  **Justification:**  No sample size specified | High risk |
| **El-Gohary et al., 2022** | Single Group pre-post test | High risk  **Justification:**  single group | High risk  **Justification:**  Outcomes primarily based on qualitative observations, non-validated measures | Low risk  **Justification:**  Intervention protocol clearly described | High risk  **Justification:**  Small sample size | High risk |
| **Elkin et al. (2022) [28]** | Single Group post test only | High risk  **Justification:**  single group | Low risk  **Justification:**  Outcomes were assessed using objective eye-tracking measures with automated data collection and predefined gaze metrics. | Low risk  **Justification:**  Intervention protocol clearly described | High risk  **Justification:**  Small sample size | Moderate risk |
| **Ip et al.,(2022) [29]** | RCT | Low risk  **Justification:**  Controlled design | Low risk  **Justification:**  Outcomes assessed using validated standardized instruments and/or objective technological measures. | Low risk  **Justification:**  Intervention protocol clearly described | Low risk  **Justification:**  Adequate sample size | Low risk |
| **Meng & Yeh (2022) [30]** | Single Group pre-post test | High risk  **Justification:**  single group | Moderate risk  **Justification:**  Outcomes included a combination of standardized rating scales and subjective reports | Low risk  **Justification:**  Intervention protocol clearly described | High risk  **Justification:**  Small sample size | Moderate risk |
| **Mojtaba Shahab et al.,**  **(2021) [31]** | Single Group pre-post test | High risk  **Justification:**  single group | Moderate risk  **Justification:**  Outcomes included a combination of standardized rating scales and subjective reports | Low risk  **Justification:**  Intervention protocol clearly described | High risk  **Justification:**  Small sample size | Moderate-High risk |
| **Johnston et al., 2020 [32]** | Single Group pre-post test | High risk  **Justification:**  single group | Moderate risk  **Justification:**  Outcomes included a combination of standardized rating scales and subjective reports | Low risk  **Justification:**  Intervention protocol clearly described | High risk  **Justification:**  Small sample size | Moderate-High risk |
| **Dixon et al., 2020 [20]** | Single Group pre-post test | High risk  **Justification:**  single group | High risk  **Justification:**  Outcomes primarily based on qualitative observations, non-validated measures | Moderate risk  **Justification:**  Intervention procedures reported but fidelity monitoring or protocol adherence not fully documented. | High risk  **Justification:**  Small sample size | High risk |
| **Herrero & Lorenzo, (2020) [33]** | RCT | Low risk  **Justification:**  Controlled design | Moderate risk  **Justification:**  Outcomes included a combination of standardized rating scales and subjective reports | Moderate risk  **Justification:**  Intervention procedures reported but fidelity monitoring or protocol adherence not fully documented. | High risk  **Justification:**  Small sample size | Moderate-High risk |
| **Junaidi et al., 2020** | Single group post test only | High risk  **Justification:**  single group | High risk  **Justification:**  Outcomes were primarily based on observational checklists, field notes, and teacher-reported questionnaires with limited standardization | Moderate risk  **Justification:**  Intervention procedures reported but fidelity monitoring or protocol adherence not fully documented. | High risk  **Justification:**  Small sample size | High risk |
| **Jyoti et al.,2020** | Quasi experimental | Moderate risk  **Justification:**  controlled cross-sectional comparison without randomization | Moderate risk  **Justification:**  structured behavioral task with objective scoring of cue-response accuracy | Moderate risk  **Justification:**  Intervention procedures reported but fidelity monitoring or protocol adherence not fully documented. | High risk  **Justification:**  Small sample size | Moderate risk |
| **Rosenfield et al., 2019 [34]** | Single group post test only | High risk  **Justification:**  single group | High risk  **Justification:**  Outcomes were primarily based on qualitative approach | Moderate risk  **Justification:**  Intervention procedures reported but fidelity monitoring or protocol adherence not fully documented. | High risk  **Justification:**  Small sample size | High risk |
| **Catalin Voss, MS et al.,**  **(2019) [35]** | RCT | Low risk  **Justification:**  Controlled design | Low risk  **Justification:**  Outcomes assessed using validated standardized instruments and/or objective technological measures | Low risk  **Justification:**  Intervention protocol clearly described | Low risk  **Justification:**  Adequate sample size | Low risk |
| **Adjorlu et al., 2018 [36]** | Single group pre-post | High risk  **Justification:**  single group | Moderate risk  **Justification:**  ecologically valid behavioral task with objective scoring, but lacking standardized validated assessment tool | Low risk  **Justification:**  Intervention protocol clearly described | High risk  **Justification:**  Small sample size | High risk |
| **Newbutt et al.,**  **2016 [37]** | Single group post only | High risk  **Justification:**  single group | Low risk  **Justification:**  Outcomes assessed using validated standardized instruments | Low risk  **Justification:**  Intervention protocol clearly described | Moderate  **Justification:**  adequate sample size but limited external validity due to single-group design and context-specific recruitment without comparison group | Moderate |
| **Beach & Wendt (2016) [38]** | Single group pre-post | High risk  **Justification:**  single group | Moderate risk  **Justification:**  semi-structured observational coding with theoretical framework support | Low risk  **Justification:**  Intervention protocol clearly described | High risk  **Justification:**  Small sample size | High risk |
| **Yufang Cheng, et al., (2015) [39]** | Single group pre-post | High risk  **Justification:**  single group | Moderate risk  **Justification:**  structured but study-specific social behavior scales with limited reported psychometric validation | Low risk  **Justification:**  Intervention protocol clearly described | High risk  **Justification:**  Small sample size | High risk |
