# Supplementary material for: Immersive wearable virtual reality for autism: a systematic review of current evidence
Source: Front Psychiatry. 2026 Jul 27;17:1771573. doi: 10.3389/fpsyt.2026.1771573 (PMC13454810; doi:10.3389/fpsyt.2026.1771573)
Supplement: Supplementary file 2 [file Table2.docx]

| **REFERENCE** | **SAMPLE** | **EXPERIMENTAL PROCEDURES**  **And DEVICE** | **SKILL AREA TARGET** | **DESIGN** | **OUTCOMES/**  **ASSESSMENT TOOLS** | **MAIN FINDING** |
| --- | --- | --- | --- | --- | --- | --- |
| **Li Li et al.,**  **(2025) [24]** | 6 children with autism age 8-14 years  Control: not applicable | **Total duration:** 1 month  **Baseline:** ABC Autism Rating Scale completed before intervention.  **Intervention:** Daily living skills training (7 skills, 2 min each) in a VR campus; facilitator guided, operator controlled VR; VR headset and controllers used; sessions recorded.  **Maintenance:** Data analyzed post-intervention to assess effectiveness.  **Device:** VIVE Pro Eye (HMD, controllers, laser localizer), SketchUp, Unreal Engine, SteamVR, cell phone camera | Daily living skills (e.g., grabbing bread, making phone calls, turning lights on/off, picking up books) | Single-group pre–post | - ABC Autism Rating Scale - Social Skills Questionnaire (SSQ) - Social Communication Questionnaire (SCQ) - Practice Interaction Success Frequency Scale - Autism Treatment Evaluation Scale (ATES) | VR intervention using a headset significantly enhanced participants’ daily living skills and overall social effectiveness |
| **Alberte Cathrine Ehrhardt Jeppesen et al.,**  **(2025) [25]** | 140 adults with autism spectrum condition (ASC) and 50 age- and gender-matched neurotypical controls | **Total duration:** Single exposure (6 scenarios, 90 s each)  **Baseline:** None reported; stationary VR observation.  **Intervention:** Participants experienced social VR scenarios with avatars of varying complexity and distractors; gaze and behavioral responses recorded.  **Maintenance:** Not reported.  **Device:** Varjo Aero HMD (eye-tracking, 200 Hz) | Social attention and gaze behaviour | Between-subjects cross-sectional design | - Gaze behavior: number of fixations, mean fixation time, dwell time on Areas of Interest (AOI) - AOI: social (head, eyes, mouth, forehead, body) vs. non-social (shopping windows, ambulance, background) - Exploratory: associations between gaze metrics and clinical, social functioning, and social cognition measures | VR-based eye-tracking provided novel insights into visual social attention in adults with ASC and may inform future clinical assessment methods |
| **Samantaray et al., 2024 [26]** | 5 children with ASD  10 neurotypical children | **Total duration:** Single-session exposure; duration not explicitly specified (task-based immersive assessment across AR and VR conditions).  **Baseline:** Not reported.  **Intervention:** Participants engaged in an **immersive Joint Attention (JA) training platform integrating Augmented Reality (AR) and Virtual Reality (VR) environments.** Tasks required participants to respond to JA cues using **eye-gaze tracking as the primary interaction modality,** focusing on shared attention and social cue following. Both behavioral responses (task performance, response latency) and physiological signals were recorded during interaction.  **Maintenance:** Not reported.  **Device:** Eye-gaze based AR/VR training system integrating immersive headset technology with physiological monitoring (specific hardware not fully detailed in the reported text). | Joint Attention, social communication, gaze-based interaction, attentional engagement | Quasi experimental design | - **Behavioral measures:** response time to JA cues (latency to register response). P - **Physiological measures:** heart rate (BPM). Comparative analysis between ASD and NT groups across AR and VR conditions | ASD participants showed significantly longer response times than NT in both AR and VR conditions. Higher BPM in ASD indicated increased physiological arousal and sensory sensitivity, suggesting interference with attentional processing. Findings highlight importance of integrating physiological monitoring in JA training systems. |
| **Kourtesis et al., (2023) [27]** | 25 adults with ASD (19 males, 6 females), aged 19–52  Control: not applicable | **Total duration:** 3 weeks **Baseline:** Neuropsychological assessment session (60 min) conducted prior to VR sessions to measure mental state/emotion recognition and executive functions (planning, working memory, inhibition) **Intervention:** 3 VR sessions per participant (1 per week) using VRESS software; each session included 5 social scenarios (gym, phone store, cinema, classroom, job interview) at a single difficulty level (easy, moderate, or difficult); participants performed scenarios in standing or sitting positions depending on scenario requirements; session duration ~60 min; demonstration on VR headset and controller usage provided at the start of each session; scenario order counterbalanced across participants; breaks provided when necessary **Post-intervention:** Participants completed questionnaires after the third VR session (Demographics and IT skills, SUTAQ, UEQ, SUS, CSQ-VR); debriefing session conducted to explain research aims **Device:** HTC Vive Pro Eye VR headset with integrated eye-tracker (120 Hz, 0.5°–1.1° accuracy); VRESS software running on SteamVR; operator-controlled interface to manage scenario difficulty, 3D character responses, gaze direction, lighting intensity, and NPC density | Social skills: emotion recognition, social interaction, planning and executive function in realistic social scenarios | Single-group pre–post | - Neuropsychological tests: Reading the Mind in the Eyes Test (mental state/emotion recognition), Tower of London (planning), Digit Recall WAIS-III (working memory), Stroop Test (inhibition) - Questionnaires: Demographics and IT skills, Service User Technology Acceptability Questionnaire (SUTAQ), User Experience Questionnaire (UEQ), System Usability Scale (SUS), Cybersickness in VR Questionnaire (CSQ-VR) | Immersive VR social skills training is feasible and well accepted in adults with ASD; performance in social scenarios predicts usability, acceptability, and functionality; planning ability predicts scenario performance; high scores on acceptability, usability, and user experience; VR interventions should be adaptive and errorless |
| **Soltiyeva et al., (2023) [28]** | 12 children with ASD (4 girls, 8 boys), aged 4–15 years;  Control: not applicable | **Total duration:** Single-session qualitative study, ~15–20 min per child.  **Pre-training:** Explanation of headset and controllers; parental consent and specialist briefing.  **Intervention:** Immersion in “My Lovely Granny’s Farm” IVRS environment; two main interactive tasks: (1) exploring the farm and greeting the virtual farmer; (2) entering the farmer’s house, interacting with virtual animals and responding to social prompts. Teacher support provided during sessions.  **Post-training:** Interviews with children and therapists; qualitative feedback on engagement, comfort, and emotional response.  **Device:** Oculus Quest 2 wireless headset with two motion controllers; Unity-based virtual environment streamed to laptop/smartphone for monitoring. | Social interaction, verbal communication, emotional engagement, and adaptation to new environments. | Single-group pre–pos**t** | - Direct behavioral observation - Qualitative interviews, and recorded video analysis by specialists - Performance scored (1 point per completed task) | 10 of 12 participants tolerated the headset on first attempt; most children interacted actively with the virtual character; improved adaptation and task performance across trials; positive engagement noted by therapists and parents. Children from ASD group 4 showed best response; minimal anxiety observed in higher-functioning participants. |
| **Alimanova et al., 2022 [29]** | 4–9 years old children with ASD diagnosis (sample size not specified)  Control: not applicable | **Total duration:** Single-session VR intervention (15–30 minutes per participant). **Baseline (Pre-assessment):** No formal baseline assessment reported. Participants were introduced to the VR environment and guided through the interaction tasks. Intervention: Immersive VR “Virtual Farm” scenario designed to promote social communication, emotional recognition, and interaction with virtual agents. The environment included: (1) exploration of the virtual farm setting, (2) interaction with a virtual farmer who adapted responses based on users’ emotional states detected through facial emotion recognition, (3) answering questions and completing interaction tasks involving virtual animals. **Feedback/Adaptation:** The virtual farmer dynamically modified interactions according to participants’ emotional responses using a facial emotion recognition system. **Post-assessment:** Behavioral observations and system-generated data (Unity Analytics) were collected to evaluate engagement, interaction, and task performance. **Device:** Oculus Quest 2 HMD; VR environment developed using Unity, Autodesk Maya, XR SDK, Face Recognition Emotion Detection SDK, and Unity Analytics. | Social communication, emotional recognition, interaction with virtual agent, task engagement | Single group posttest only | - Observational checklist - emotional response tracking (system-based facial emotion recognition) - task completion data - qualitative expert evaluation | Results showed high engagement and active participation in most children. VR environment increased motivation, exploration, and social interaction with virtual agent. System considered promising for ASD rehabilitation with high interactivity and acceptability. |
| **El-Gohary et al., 2022 [30]** | 11 children and adolescents with ASD (age 7–18 years).  Control: not applicable | **Total duration:** Multi-session intervention (VR training + pre/post real-world assessment in hospital cafeteria). **Baseline (Pre-assessment):** Shopping task in real cafeteria (select items, ask price, pay). **Intervention:** VR serious game simulating hospital cafeteria with 4 progressive levels: (1) free navigation training, (2) item selection from shopping list using Leap Motion, (3) payment task with virtual money selection, (4) full integrated shopping scenario with social interaction (avatars + sound environment). **Feedback**: immediate reinforcement (sounds, color feedback, avatar speech). **Post-assessment:** Repetition of shopping task in real cafeteria to evaluate transfer.  **Device:** Oculus Rift HMD; Leap Motion hand-tracking sensor; Unity 3D + SketchUp; laptop (Windows 10, i7, 16GB RAM, NVIDIA 2060). | Daily living skills – shopping skills (item selection, price understanding, payment execution, social interaction with cashier). | Single-group pre–post design | - Task success rate (Effectiveness = successful tasks / total tasks ×100) - Task completion time (minutes) - Pre- vs post- performance comparison in real cafeteria - Observational behavioral measures during VR training | VR training improved shopping-related functional skills in ASD participants. Only 6 participants completed full protocol. VR environment increased engagement and task learning. Skills acquired in VR showed transfer to real-world cafeteria tasks. Leap Motion and gamification improved usability and motivation. |
| **Elkin et al. (2022) [31]** | 10 children/youth with ASD, ages 6–17;  Control: not applicable | **Total duration:** 1 session per participant (~10 minutes each) **Baseline:** Participant orientation and avatar customization; brief familiarization with HTC Vive Pro Eye VR headset to ensure comfort **Intervention:** Single immersive VR social-skills session in a virtual classroom; participants responded to 5 questions posed by a virtual avatar while eye-tracking data were collected; environment included classroom objects (desk, globe, whiteboard, chairs) **Control:** Not applicable (pilot case-series; no control group) **Post-intervention:** Analysis of gaze fixation and visual search behaviors from VR session; classification of gaze on avatar’s eyes, face, mouth, hands, and environmental objects; comparison across ASD severity levels **Device:** HTC Vive Pro Eye headset with Tobii integrated eye-tracking; Alienware Area 51 laptop running VR SAFE software via Steam; VR environment created specifically for the study (virtual classroom), eye-tracking data logged in real-time | Social skills, affective expression, gaze fixation, visual attention | Single-group posttest-only | Eye-tracking metrics (number and duration of gaze on avatar’s eyes, face, mouth, hands, and environmental objects); participant demographics (age, sex, ASD severity) | Differential gaze fixation and visual search patterns observed across mild, moderate, and severe ASD; eye-tracking may provide objective metric of social-skills training effectiveness |
| **Ip et al.,(2022) [32]** | EG:48 (41 M;7 F) mean age: 8 years  CG:59 (47 M; 12 F) mean age:9 years | **Total duration:** 15 weeks  **Baseline:** Pre-assessment 2 weeks before first session using PEP-3 subtests (Affective Expression, Social Reciprocity) and Raven’s Progressive Matrices for non-verbal IQ  **Intervention:** 2 VR sessions/week at schools (30 sessions total); each session 60 min (15 min briefing, 30 min VR exposure, 15 min debriefing); groups of 4 children per session; VR scenarios based on Kolb’s experiential learning model; facilitation by trained research team members and schoolteachers  **Control:** Waitlist; no VR intervention during study period  **Post-intervention:** Re-assessment within 2 weeks after last session using same measures; observational data, teacher and trainer notes collected  **Device:** Oculus Rift VR headset with Touch controllers, Lenovo Legion Y520 laptop (GTX 1060 GPU, 16GB RAM), sensors for 6-DoF tracking; VR content created in Blender and Unity | Affective expression; social reciprocity | RCT | - PEP-3 subtests (Affective Expression, Social Reciprocity) - Raven’s Progressive Matrices - observational data during VR sessions - trainer and teacher notes | VR-enabled school-based intervention significantly improved affective expression and social reciprocity in children with ASD compared to control; demonstrated feasibility and sustainability of VR delivery in school setting |
| **Meng & Yeh (2022) [33]** | 10 high-functioning autistic students  Age 6-13y  Control: not applicable | **Total duration:** 8 weeks  **Pre-intervention:** Familiarization with VR, needs assessment, and completion of teacher/parent questionnaires  **Intervention:** VR social skills course with four scenarios (“Classroom,” “Exhibition,” “Restaurant,” “Park”); 10-min VR sessions per class preceded by 25-min preparatory activities (picture book + course introduction); 1–2 sessions/week  **Post-intervention:** Re-administration of questionnaires, parent and teacher interviews, observation of student performance  **Device:** Head-mounted VR display with handheld controllers; virtual environments based on “One Art Tour with Students” | Social skills: interpersonal behaviors, environmental adaptability, communication, emotional management | Single-group pre–post | - Elementary and Junior High School - Students Social Skills Behavior Scale (teacher-rated) - Social Skills Effectiveness Survey (parent-rated) - teacher/parent interviews - observational data during VR sessions | VR intervention improved students’ inappropriate social behaviors and environmental adaptability; parents and teachers reported enhanced social skills; minor technical/operational issues identified (e.g., VR display problems, controller handling) |
| **Mojtaba Shahab et al.,**  **(2021) [34]** | 5 male children with high-functioning ASD, age 6–8 years  Control: not applicable | **Total duration:** 8–10 weeks  **Baseline:** Pre-test and practice with virtual robot and instruments  **Intervention:** Weekly 15–20 min VR sessions; children learned 7 musical notes, imitated virtual robots, and practiced simple-to-complex melodies; performance automatically assessed and video recorded.  **Maintenance:** Follow-up session 2 months post-intervention.  **Device:** HTC Vive, Virtual robot (Nima), xylophone, video camera, screen recording | Musical skills (note recognition, melody performance), cognitive skills, imitation, attention, motor coordination | Single-group pre–post + follow-up | - examining the children’s ability to recognize/express colors, - performing the Stambak Rhythmic Structures Reproduction Test to assess the children’s musical skills - a quantitative/qualitative evaluation of the children’s social and cognitive skills, focusing on imitation skills and joint attention (extracting items from the Early Social Communication Scales (ESCS)   Four questionnaires:   - Autism Social Skills Profile (ASSP) - Gilliam Autism Rating Scale (GARS) - Autism Checklist - Parenting Stress Index-Short Form (PSI-SF) were completed by the parents at several stages (before and after the treatment period). | Participants showed a general improvement in musical skills across the intervention sessions. Slight cognitive gains were observed, likely limited by a ceiling effect. The VR-based music education program was feasible and effective for children with high-functioning autism. |
| **Johnston et al., 2020 [35]** | 6 children with ASD  4 males  2 females  16–19 years  Control: not applicable | **Total duration:** 4 weeks  **Baseline:** None reported; initial probe of anxiety to target sounds.  **Intervention:** Weekly 30-min sessions with SoundFields game; 20 exposures per session, progressively closer; minimal support and praise.  **Maintenance:** Post-session questionnaires assessed anxiety.  **Device:** Oculus Rift CV1 | Desensitization to auditory stimuli  Sensory domain | Single-group pre-post | - Modified Smiley-Face Assessment Scale - Tracked Voluntary Participant Interaction with Target Auditory Stimuli | All participants experienced a decrease in their SUD following the 4 week intervention. The average decrease in self-reported anxiety was 1.7 points, with a minimum decrease of 1 point and a maximum of 3 points. |
| **Dixon et al., 2020 [20]** | 3 participants, all male, aged 4–10 years.  Control: not applicable | **Total duration:** 2–3 days  **Baseline:** Real streets probes asking “Is it safe to cross?”  **Intervention:** VR training with short street videos, 3–5 min per session, flexible prompt fading; reinforcement with verbal praise, tokens, or preferred activities; distractions added in later phases  **Maintenance:** Generalization tested via additional VR training with distractors and longer videos  **Device:** Oculus Rift | Daily living / safety skills — specifically, street-crossing safety and hazard recognition. | Single-group pre–post + generalization | Data was collected using a paper data sheet. Participants’ responses for each applicable step were scored as correct, incorrect, or prompted. For each session, data were summarized by calculating the percentage correct across applicable steps. | After modification to the VR training environment, all 3 participants reached mastery criteria in both VR and natural environment settings. |
| **Herrero & Lorenzo, (2020) [36]** | CG: 7  EG: 7  Range age: 8-15 years | **Total duration:** 10 sessions during school holidays **Baseline:** 1 month pre-intervention familiarization with HMD and virtual environments; needs assessment; scenario scripting by special education teachers; pre-intervention questionnaires completed by parents, therapists, and researcher **Intervention:** Immersive VR sessions in classroom and garden scenarios; participants practiced scripted social and emotional tasks including verbal/non-verbal communication, joint attention, empathy, theory of mind (ToM), and emotional regulation; sessions observed and guided by researchers **Control:** No intervention administered to control group **Post-intervention:** Re-administration of questionnaires to parents, therapists, and researcher; performance evaluation via VR system metrics (avatar interactions, visual contact duration, researcher-triggered actions) and observational data | Social and emotional skills | RCT | - Data collected from the general questionnaires - Data about the students’ performance during the sessions - Data automatically registered by the system. | IVR intervention produced measurable improvements in social and emotional competences in the study group, including communication, joint attention, empathy, emotional regulation, and theory of mind; VR metrics and questionnaires indicated transfer and acquisition of targeted skills, while the control group showed no significant change |
| **Junaidi et al., 2020 [37]** | 7 autistic children, all men 13-18 years  Control: not applicable | **Total duration:** not clearly specified (two VR trial sessions, adaptation + instruction phase). **Intervention:** VR learning product developed using Borg & Gall R&D model; Unity-based VR application with adjustable image quality (low–high). Tasks included instructional learning activities evaluated via HMD usability, object readability, and comprehension of instructions. Teacher validation included (n=4 experienced special education teachers). **Device:** HMD (not specified model), VR application developed in Unity™ | Educational learning skills; VR usability and comprehension; instructional understanding; attention to VR tasks | Single group posttest only | ● Observation checklists (HMD usability, object readability, instruction comprehension) ● Field notes ● Teacher evaluation questionnaires (validity, usability, usefulness, instruction suitability) ● Percentage-based effectiveness criteria | VR content was rated as **moderately effective to valid (82.9%)**. Most participants showed initial anxiety (71.4%) and surprise (57.1%) when using HMD, with fatigue reported in 71.4%. Object readability was high, but instruction comprehension was less effective (only 28.6% responded appropriately). Teacher evaluation indicated high validity, although ergonomic issues (visual obstruction, neck discomfort) and usability limitations were noted. Overall, VR was considered valid and potentially useful for educational purposes but requires refinement in instruction design and ergonomic usability. |
| **Jyoti et al.,2019 [38]** | 20 ASD participants 20 age-matched typically developing (TD) participants | **Total duration:** Not explicitly reported; task-based VR Joint Attention training with repeated cue exposure within a single experimental session.  **Baseline:** Not reported.  **Intervention:** Participants used a **virtual reality (VR) Joint Attention training platform** designed to assess and train JA skills through a **hierarchical prompting system**. The system presented progressively supportive social cues, including **eye gaze, head turn, finger pointing, and visual “sparkling” cues**, with adaptive adjustment of prompt level based on participant performance. Tasks required users to respond to joint attention cues with increasing or decreasing assistance depending on accuracy and responsiveness.  **Maintenance:** Not reported.  **Device:** VR-based Joint Attention training system (head-mounted VR platform; specific hardware not explicitly reported in the provided description). | Joint Attention, social cue detection, gaze following, social communication | Quasi experimental design | Performance-based measures:   - ability to respond to different JA cues (eye gaze, head turn, pointing, visual cue hierarchy); - cue hierarchy success rate | ASD participants showed impairments in following eye-gaze cues but better performance with more explicit cues (e.g., finger pointing and sparkling cues). TD participants successfully responded to eye-gaze cues without additional prompting, indicating clear group differences in JA processing. The system effectively identified JA deficits and demonstrated feasibility of adaptive VR-based training. |
| **Rosenfield et al., 2019 [39]** | 2 children with ASD  2 children in a technology probe: 1 female child with ASD (6 years old) and 1 male child with ADHD (7 years old), accompanied by their mothers  Control: not applicable | **Total duration:** Single exploratory session (15 min) **Baseline:** None reported **Intervention:** Children navigated a virtual shop, interacted with a virtual shopkeeper; joint attention, verbal and non-verbal communication assessed via observation and interviews **Maintenance:** Not reported **Device:** Oculus Rift VR headset | social communication | Single-group posttest-only | - Qualitative approach - Interview | The VR environment was feasible, engaging, and acceptable for neurodiverse children; promoted conversational practice and social skill engagement, but efficacy not formally tested due to small sample size |
| **Catalin Voss, MS et al.,**  **(2019) [40]** | EG: 40 (37 M; 3 F) mean age 8 years    CG:31 (16 M; 15 F) mean age 8 years | **Total duration:** 6 weeks **Baseline:** Pre-test with ABA therapy only **Intervention:** 20-min home sessions, 4 times/week using Superpower Glass wearable + ABA (treatment) or ABA alone (control) **Maintenance:** Not explicitly reported **Device:** Superpower Glass wearable, smartphone app | Socialization, facial engagement, and emotion recognition | RCT | - The SRS-II - VABS-II - Nepsy-II - Emotion Guessing Game (EGG) - Social Communication Questionnaire | Wearable AI-driven Superpower Glass intervention significantly improved socialization in children with ASD, comparable to gains with standard care; reinforces facial engagement and emotion recognition as potential mechanisms |
| **Adjorlu et al., 2018 [41]** | 5 children with ASD  Age ranged from 18 to 22 years old  Control: not applicable | **Total duration:** Not explicitly stated (5 sessions implied) **Baseline:** Pre-VR assessment of money-handling skills with real coins and bills **Intervention:** VR money training in a virtual park; 3 levels: match images, match numeric values, practice purchases; HTC Vive headset and controllers used **Maintenance:** Not explicitly reported **Device:** HTC Vive VR system (headset + controllers) | money management skills  daily living skills | Single-group pre–post | - Pre- and post-VR training measurements of the participants’ money skills was conducted - Counting the number of correct purchases done by the students using real money - Pre post VR, training measurement of the participant money skills was conducted | Four out of the five participants showed some improvement in their money skills after five training sessions with the VR application. |
| **Newbutt et al.,**  **2016 [42]** | 29 children with ASD  22 males  7 females  Control: not applicable | **Total duration:** Phase I: 10 min; Phase II: 25 min per participant **Baseline:** Not explicitly reported **Intervention:** Phase I: VR cinema, café, safari. Phase II: Apollo 11 mission, Tuscan house; participants engaged in navigation, social cues, and environmental interactions; verbal and non-verbal responses noted. **Maintenance:** Not reported **Device:** Oculus Rift, headphones, Xbox 360 controller, laptop | Spatial presence and immersion, basic social interaction, environmental navigation, sustained attention and engagement. | Single-group posttest-only | - The Independent Television Commission-Sense of Presence Inventory (ITC-SoPI) | From behavioral observations and self-reported questionnaires revealed that people with ASD generally accepted the HMD and were willing to complete the tasks associated with VR scenarios in full. |
| **Beach & Wendt (2016) [43]** | 2 students with ASD: John (15 y/o, high-functioning), Sam (18 y/o, high-functioning/low-functioning); participants selected from summer camp attendees  Control: not applicable | **Total duration:** 4 weeks  **Baseline:** Needs assessment and scenario scripting by special education teachers  **Intervention:** 2 hours/day practicing scripted social interaction scenarios in immersive VR environment (IVE) at university + 3 hours/day practicing similar scenarios in real-world summer camp, 5 days/week  **Maintenance:** Transfer of skills assessed in real-world scenarios; follow-up during camp sessions  **Device:** Head-mounted VR unit with full visual immersion, surround sound, and haptic feedback; virtual environments designed in Second Life | Social interaction skills | Single-group pre–post + transfer | - Observations - Scenario transfer to real-world settings - Coding based on Social Skills Menu (Baker, 2007) - Performance improvement analysis using HPT framework | Both participants demonstrated improvement in social interaction skills within the IVE, with observable transfer and maintenance of skills in corresponding real-world situations |
| **Yufang Cheng, et al., (2015) [44]** | 3 children with ASD  Age: 10–13  Control: not applicable | **Total duration:** 6 weeks **Baseline:** 3 baseline sessions per participant **Intervention:** 5 intervention sessions per participant, 30–40 min each; participants used immersive HMD to navigate 3D environments (bus stop, bus ride, street scenes, classroom); responses coded by researchers; reinforcement given **Maintenance:** 3 maintenance sessions post-intervention **Device:** Head-mounted display (immersive VR system) | Social understanding and social skills | Single-group pre–post + maintenance | - Social events card (SEC) - Social behaviors scale (SBS) | Targeted social behaviors improved from baseline to intervention and were maintained, suggesting VR may be an effective tool for promoting social understanding in ASD. |
